# Concurrent utilisation of cancer screening tests in the general population aged 50–69: an analysis of the 2017 Swiss Health Survey

**DOI:** 10.3389/ijph.2026.1609624

**Published:** 2026-06-23

**Authors:** Audrey Butty Dettwiler, Jessica Nakiyingi-Miiro, Jean-Luc Bulliard

**Affiliations:** 1 Department of Epidemiology and Health Systems, Unisanté, University Center for Primary Care and Public Health and University of Lausanne, Lausanne, Switzerland; 2 London School of Hygiene and Tropical Medicine, London, United Kingdom

**Keywords:** cancer screening, concurrent utilisation, factors, population-based, screening uptake

## Abstract

**Objectives:**

This study assessed the extent and factors of concurrent utilisation of cancer screenings in Switzerland.

**Methods:**

Data from the 2017 Swiss Health Survey, representing 1,091,813 females and 1,072,940 males aged 50–69, were analysed. Weighted descriptive analyses estimated sex-specific proportions of individuals concurrently up to date with recommended screening for cervical, breast, colorectal, prostate and skin cancer. Multivariable binomial weighted logistic regressions examined associations between concurrent utilisation and sociodemographic, lifestyle, health, health services utilisation, security and social support factors.

**Results:**

Approximately one in three Swiss residents were concurrent screening users. Concurrent utilisation increased with age and frequent medical visits. Among females, concurrent utilisation was positively associated with living in French- or Italian-speaking Switzerland and having tertiary education, and negatively associated with being divorced, separated or widowed, and with non-European origin. Among males, concurrent utilisation was positively associated with income, and negatively associated with unhealthy lifestyles, low health concern, and higher health insurance deductibles.

**Conclusion:**

Further research is needed to clarify the factors underlying differences in screening behaviours. Given the temporal instability of these behaviours, the findings should be interpreted cautiously.

## Introduction

The health and economic burden associated with cancer, such as potential years of life lost (PYLL), loss of productivity, or costs of treatment, represents a major public health problem in many countries [1–3].

In Switzerland, cancer accounts for the highest number of PYLL before age 70 [4](p.17) and is the second leading cause of death [5]. Prostate and breast cancers are the most commonly diagnosed cancers among men and women, respectively, followed by lung cancer, colorectal cancer, and melanoma [6]. Cervical cancer, although less common, remains a health concern, particularly among women who do not participate in Human Papillomavirus (HPV) vaccination and screening. While age-standardised incidence rates for most cancers are expected to stabilise by 2025, a large increase in the number of new cases across all cancer types is predicted, driven by population aging and growth [7].

Screening is an effective means to reduce the burden of several common cancers [8–12]. Current screening recommendations for the most common cancer types in Switzerland are outlined hereafter. For breast cancer, biennial mammography screening is recommended for women aged 50–74 [13]. Colorectal cancer screening is recommended for women and men aged 50–74, using either colonoscopy every 10 years or biennial faecal occult blood test (FOBT) [14]. Cervical cancer screening with a Papanicolaou (Pap) smear is recommended from age 21, and HPV testing from age 30, with screening continued every 3 years until age 70 [15, 16]. For prostate cancer screening, there is no consensus in Switzerland; however, some experts and the Swiss Urology Society recommend offering PSA-guided screening to men aged 50–69 within a shared decision-making process [17, 18], in line with the recommendations of the European Association of Urology [19]. The recommended frequency of screening varies according to risk category but is 2 years for most individuals [20, 21]. Lung cancer screening is recommended with a LDCT for high-risk individuals [22]. Despite its high incidence of melanoma,4(p.43), [23] there is no recommendation for skin cancer screening in Switzerland. However, it has participated in the annual Euromelanoma campaigns since their inception, with a National Skin Cancer Screening Day [24, 25].

In Switzerland, not all recommended screening tests are necessarily reimbursed [26]. The costs of tests for the early detection of cancer in the general population (not high-risk groups) are covered by the universal Swiss health insurance under specific conditions: two annual Pap smear for cervical cancer screening are covered for women aged 21–70, and then in case of normal results, every 3 years; a mammography as part of an organised programme from age 50 is covered without deductible every 2 years, and a colonoscopy or a FOBT between age 50–74 (50–69 at the time of the study) are covered every 10 or 2 years, respectively (if it is part of an organised cantonal programme, no deductible is charged) [27]. (art. 12e) An opportunistic mammography for breast cancer screening is thus not covered (except for high-risk women) while colorectal cancer screening is covered when opportunistic or organised. Population-based, regional programmes exist only for breast and colorectal cancer screening, and participation in these organised programmes is recommended over opportunistic screening. However, as healthcare is primarily organised and delivered by the cantons (i.e., administrative states), the availability of breast and colorectal screening programmes varies from canton to canton [28]. Thus, for most types of cancer, screening occurs opportunistically in the general population.

Studies, in Switzerland and beyond, have found numerous factors associated with cancer screening utilisation, including socioeconomic factors [29–36], ethnic or racial background [29, 36], living in rural areas [30, 32], health behaviours [37], health services utilisation [32, 35], and health insurance coverage [34, 38]. Current evidence on cancer screening behaviour also supports the hypothesis of complementarity between cancer screenings, with individuals undergoing one type of screening being more likely to participate in other cancer screenings [32, 37, 39–42].

Fewer studies have examined the use of concurrent (i.e., multiple) cancer screenings [41, 43–49], reporting wide variations in the proportion of individuals using multiple screenings. Non-utilisation of concurrent cancer screenings has been reported to be associated with socioeconomics factors, such as low education, high social deprivation [45–48], and ethnic background [49], as well as low self-rated health status [45], albeit the association of comorbidities with concurrent utilisation of cancer screenings appears unclear [47, 48]. Overall, these social inequalities in preventive health-seeking behaviours contribute to broader social health inequalities.

However, practices of cancer screening differ considerably between countries. In Switzerland, studies have reported temporal and mainly regional variations in utilisation of breast, colorectal and prostate cancer screenings [50–52]. However, to date, no information is available on the extent and factors associated with concurrent screening among Swiss residents.

Using data from the nationwide 2017 Swiss Health Survey (SHS), we aimed to determine the proportion of concurrent utilisation of cancer screenings and identify factors associated with it. Understanding patterns of multiple screening could identify gaps in preventive care delivery and population groups who may benefit from targeted interventions, guiding the development of effective strategies to optimise cancer screening utilisation and ultimately reduce social health inequalities.

## Methods

### Data source

The SHS is a cross-sectional, nationwide, population-based quantitative survey conducted every 5 years by the Federal Statistics Office (FSO), using a random sample of the permanent resident population aged 15 [53]. The FSO stratifies the sample by canton and weights the results by region of residence, age, sex, nationality, marital status, and household size in order to have a representative sample of the Swiss population across all linguistic regions. Information is collected through a telephone interview or, in case of difficulties to answer, through a face-to-face or a proxy interview, followed by a written questionnaire (except for proxy interviews). In 2017, 22,134 of 43,769 (51%) contacted individuals participated in a telephone (21,205), face-to-face (20) or proxy (909) interview between 16 January and 22 December 2017. Of the 21,225 individuals invited to fill the written questionnaire, 18,832 (89%) completed it. The interview and written questionnaires are available on the FSO website [54].

The analysis was restricted to participants aged 50–69 who completed both the telephone interview and written questionnaire. This restriction ensured alignment with the target age range of most cancer screening guidelines [55], allowed inclusion of security support data (available in the written questionnaire), and minimised potential bias from face-to-face or proxy interviews.

### Concurrent utilisation of cancer screenings

The 2017 SHS collected screening information for breast, colorectal, prostate, cervical and skin cancers. In order to target the profiles of regular users more accurately, the utilisation of a cancer screening was defined as the last self-reported preventive test performed within recommended intervals in Switzerland: a mammography within the past 2 years for breast cancer [13]; a colonoscopy or faecal occult blood test (FOBT) within the past 10 or 2 years for colorectal cancer [14]; a prostate-specific antigen (PSA) test or digital rectal exam within the past 2 years for prostate cancer [17, 33]; a Pap test within the past 3 years for cervical cancer [15]. As there is no recommendation about frequency of visual inspection for skin cancer in the Swiss general population, a limit was set arbitrarily at 5 years.

In the absence of a standard definition, concurrent utilisation was defined as the maximum number of recommended screening tests minus one, avoiding overly restrictive criteria given the lack of formal skin cancer screening recommendations. Concurrent utilisation is thus a proxy for multiple utilisation patterns. For females, the primary outcome measure was concurrent utilisation of breast, colorectal, cervical, and skin cancer screenings (two or fewer screenings as reference; three or four screenings as concurrent utilisation). For males, the primary outcome measure was concurrent utilisation of colorectal, prostate, and skin cancer screenings (one or fewer screenings as reference; two or three screenings as concurrent utilisation).

### Statistical analyses

Five sets of covariates, identified from the available literature as potentially associated with cancer screening utilisation, were included: sociodemographic, lifestyle, health, health services utilisation, and social security and support factors.

All analyses were performed separately by sex assigned at birth (female/male). Age was centred on the mean, and other continuous variables were categorised according to their distribution. For each covariate, missing values (22.8% and 18.7% in total across all covariates for females and males, respectively) were included as a separate category (not displayed) in multivariable analyses.

Multicollinearity between all pairs of variables was assessed using Spearman rank correlation test (cut-off 0.5) before model fitting. When two highly correlated variables measured the same underlying dimension, the most informative variable (e.g., number of medical visits rather than having ever had one medical visit in the past year) was retained in multivariable analyses to keep models parsimonious and maximise statistical power.

The sampling weight of the written survey, as recommended by the FSO, was applied in all analyses to ensure proportional representation of the Swiss population in terms of region of residence, age, sex, and socioeconomic status[53] (p.20). Descriptive statistics are presented as unweighted counts, weighted counts, and weighted percentages with 95% confidence intervals (CI).

Multivariable binomial weighted logistic regression models were first performed separately for each set of covariates to examine the association between concurrent utilisation and each set-specific covariates. For each set-specific model, a backward stepwise selection procedure was applied. Variables showing a significant association with the outcome at the Akaike information criterion threshold (p < 0.157) [56–58] were retained in the set-specific model. For each variable removed by this procedure, a Wald test was performed to assess whether its inclusion would improve model fit. Variables showing a significant improvement (p < 0.05), were retained in the set-specific model.

All variables retained in the set-specific models were included to build a final combined model encompassing the five set of covariates. Multivariable binomial weighted logistic regression models were performed separately for females and males to examine the associations between concurrent utilisation and each covariate, following the same methodology described above for the set-specific models. In addition, all variables removed at the initial step (set-specific models) were reassessed using a Wald test, and included in the final model if they showed a significant effect [56]. For each model, adjusted odds ratios (aORs) and 95% CI were calculated. Data were analysed using Stata 17.

## Results

Three thousand four hundred and thirty-six females and 3,081 males aged 50–69 completed both the telephone interview and written questionnaire, representing 1,091,813 and 1,072,940 Swiss female and male residents, respectively. Table 1 summarises the characteristics of each target population. The most represented categories were being aged 50–54, married, employed and Swiss, having achieved secondary education and an annual deductible of 300 Swiss Francs (CHF), and living in German-speaking Switzerland, in an urban area, as a couple without children. Most females and males were relatively healthy, which was consistent with the higher proportion of individuals who reported paying attention to their lifestyle to maintain health and who perceived their health as very good or good. Most females and males reported having had at least one medical visit and no hospitalisation in the past year, as well as moderate or high social support. Differences between the sexes were also observed. The most frequently reported equivalised disposable income (EDI) (i.e., net monthly income adjusted for household size) was CHF 3,000–4,499 among females and CHF 6,000 or higher among males (1 CHF ≈1.07 Euro ≈1.25 US Dollar as of January 2026). Females more frequently reported consuming 3-4 portions of fruit and/or vegetables per day, whereas intake was lower among males. Most females had a normal weight, whereas the most represented category among males was overweight.

**TABLE 1:** Characteristics of female and male participants aged 50–69 (Swiss Health Survey, Switzerland, 2017).

​	Females				Males			
​	Missing values	Unweighted count	Weighted count	Weighted % (95% CI)	Missing values	Unweighted count	Weighted count	Weighted % (95% CI)
​	​	3,436	1,091,813	​	​	3,081	1,072,940	​
Socio-demographics								
Age groups	0	​	​	​	0	​	​	​
* 50–54 years*	​	960	327,494	30.0 (28.2–31.9)	​	965	348,759	32.5 (30.5–34.5)
* 55–59 years*	​	884	277,062	25.4 (23.7–27.2)	​	778	277,242	25.8 (24.0–27.7)
* 60–65 years*	​	849	260,360	23.8 (22.2–25.6)	​	716	252,619	23.5 (21.8–25.4)
* 65–69 years*	​	743	226,898	20.8 (19.2–22.4)	​	622	194,320	18.1 (16.6–19.7)
Education^1^	9	​	​	​	5	​	​	​
* Primary*	​	600	193,443	17.8 (16.3–19.4)	​	292	111,369	10.4 (9.1–11.8)
* Secondary*	​	1,989	621,870	57.2 (55.2–59.1)	​	1,445	495,886	46.3 (44.2–48.4)
* Tertiary*	​	838	272,746	25.1 (23.4–26.9)	​	1,339	464,040	43.3 (41.3–45.4)
Marital status	0	​	​	​	0	​	​	​
* Single*	​	330	113,584	10.4 (9.2–11.7)	​	312	132,009	12.3 (10.9–13.9)
* Married*	​	2,285	686,316	62.9 (60.9–64.8)	​	2,318	755,242	70.4 (68.3–72.4)
* Divorced or separated*	​	604	223,471	20.5 (18.8–22.2)	​	407	170,327	15.9 (14.3–17.6)
* Widowed*	​	217	68,441	6.3 (5.4–7.3)	​	44	15,362	1.4 (1.0–2.0)
Occupation	2	​	​	​	2	​	​	​
* Economically inactive*	​	1,258	377,894	34.6 (32.8–36.5)	​	693	234,205	21.8 (20.2–23.6)
* Unemployed or homemaker*	​	42	15,581	1.4 (1.0–2.1)	​	48	19,780	1.8 (1.3–2.6)
* Employed*	​	2,134	697,446	63.9 (62.0–65.8)	​	2,338	818,209	76.3 (74.5–78.0)
Nationality	0	​	​	​	0	​	​	​
* Swiss*	​	3,049	911,708	83.5 (81.8–85.1)	​	2,555	847,387	79.0 (77.1–80.7)
* Northern and western European*	​	133	66,636	6.1 (5.0–7.4)	​	185	90,873	8.5 (7.2–9.9)
* South-western European*	​	174	73,534	6.7 (5.7–7.9)	​	226	81,401	7.6 (6.6–8.7)
* Eastern and south-eastern European*	​	63	32,796	3.0 (2.3–3.9)	​	91	42,150	3.9 (3.2–4.8)
* Non-European*	​	17	7,139	0.7 (0.4–1.1)	​	24	11,129	1.0 (0.7–1.6)
Linguistic region	0	​	​	​	0	​	​	​
* German-speaking*	​	2,283	786,665	72.1 (70.5–73.5)	​	2,111	788,378	73.5 (71.9–75.0)
* French-speaking*	​	885	253,491	23.2 (21.8–24.7)	​	741	236,147	22.0 (20.5–23.6)
* Italian-speaking*	​	268	51,657	4.7 (4.2–5.4)	​	229	48,415	4.5 (3.9–5.2)
Region of residence^2^	0	​	​	​	0	​	​	​
* Urban*	​	1,922	652,735	59.8 (57.8–61.7)	​	1,707	648,430	60.4 (58.4–62.4)
* Dense suburbs and rural centres*	​	774	240,350	22.0 (20.4–23.7)	​	717	236,713	22.1 (20.4–23.8)
* Rural*	​	740	198,728	18.2 (16.8–19.7)	​	657	187,797	17.5 (16.1–19.1)
Household type	0	​	​	​	1	​	​	​
* Single individual*	​	734	264,441	24.2 (22.5–26.1)	​	467	200,025	18.6 (17.0–20.5)
* Couple without children*	​	1,625	487,369	44.6 (42.7–46.6)	​	1,379	458,447	42.7 (40.7–44.8)
* Couple with children*	​	796	246,903	22.6 (21.0–24.3)	​	1,075	352,696	32.9 (31.0–34.8)
* Single parent with children*	​	224	75,205	6.9 (5.9–8.0)	​	90	35,427	3.3 (2.6–4.3)
* Multi-family household or non-family household with several individuals*	​	57	17,895	1.6 (1.2–2.2)	​	69	26,193	2.4 (1.9–3.2)
Equivalised disposable income^3^	360	​	​	​	210	​	​	​
* < CHF 3,000*	​	638	205,954	21.2 (19.5–23-0)	​	473	160,257	16.1 (14.6–17.8)
* CHF 3,000–4,499*	​	976	300,522	31.0 (29.1–33.0)	​	808	262,960	26.5 (24.7–28.4)
* CHF 4,500–5,999*	​	679	212,667	21.9 (20.2–23.7)	​	661	224,605	22.6 (20.9–24.5)
* ≥ CHF 6,000*	​	783	251,031	25.9 (24.1–27.8)	​	929	344,548	34.7 (32.7–36.8)
Lifestyle								
Physical activity^4^	74	​	​	​	​	​	​	​
* Inactive*	​	247	80,592	7.6 (6.5–8.7)	42	249	91,787	8.7 (7.6–10.0)
* Partially active*	​	552	178,121	16.7 (15.2–18.3)	​	495	163,207	15.5 (14.0–17.0)
* Sufficiently active*	​	2,563	807,652	75.7 (74.0–77.4)	​	2,295	800,524	75.8 (74.0–77.6)
Fruit and/or vegetables consumption	12	​	​	​	18	​	​	​
* < 5 days per week*	​	161	53,672	4.9 (4.1–5.9)	​	401	144,422	13.5 (12.1–15.1)
* 0–2 portions per day ≥ 5 days per week*	​	902	296,674	27.3 (25.5–29.1)	​	1,329	459,964	43.1 (41.0–45.2)
* 3–4 portions per day ≥ 5 days per week*	​	1,287	401,925	36.9 (35.0–38.9)	​	933	326,415	30.6 (28.7–32.6)
* ≥5 portions per day ≥ 5 days per week*	​	1,074	335,502	30.8 (29.0–32.7)	​	400	136,234	12.8 (11.5–14.2)
Alcohol consumption^5^	2	​	​	​	0	​	​	​
* Abstainer*	​	575	191,695	17.6 (16.1–19.2)	​	287	116,452	10.9 (9.5–12.3)
* Low risk*	​	2,703	855,232	78.4 (76.7–80.0)	​	2,618	894,393	83.4 (81.6–84.9)
* Moderate or high risk*	​	156	44,550	4.1 (3.4–4.9)	​	176	62,096	5.8 (4.8–6.9)
Smoking status	1	​	​	​	0	​	​	​
* Never*	​	1,708	544,200	49.9 (47.9–51.9)	​	1,244	420,451	39.2 (37.2–41.2)
* Ex-smoker*	​	949	295,478	27.1 (25.3–28.9)	​	1,017	354,387	33.0 (31.1–35.0)
* Smoker*	​	778	251,854	23.1 (21.4–24.8)	​	820	298,103	27.8 (25.9–29.7)
*Health*								
Body mass index^6^	26	​	​	​	13	​	​	​
* Underweight*	​	137	39,680	3.7 (3.0–4.4)	​	10	3,438	0.3 (0.1–0.7)
* Normal weight*	​	1,889	593,967	54.8 (52.8–56.8)	​	1,186	421,940	39.5 (37.4–41.5)
* Overweight*	​	942	301,975	27.9 (26.1–29.7)	​	1,379	469,160	43.9 (41.8–46.0)
* Obese*	​	442	148,480	13.7 (12.3–15.2)	​	493	174,847	16.4 (14.8–18.0)
Chronic disease or long-term health problem	11	​	​	​	7	​	​	​
* No*	​	2,011	626,173	57.5 (55.5–59.5)	​	1,907	649,575	60.7 (58.6–62.7)
* Yes*	​	1,414	462,082	42.5 (40.5–44.5)	​	1,167	420,792	39.3 (37.3–41.4)
Cancer in the past year	3	​	​	​	5	​	​	​
* No*	​	3,363	1,070,841	98.2 (97.6–98.6)	​	3,004	1,045,936	97.7 (97.0–98.2)
* Yes*	​	70	19,697	1.8 (1.4–2.4)	​	72	24,604	2.3 (1.8–3.0)
Self-reported health	2	​	​	​	3	​	​	​
* Very good or good*	​	2,797	881,095	80.7 (79.1–82.3)	​	2,519	868,718	81.1 (79.3–82.7)
* Medium*	​	497	162,633	14.9 (13.5–16.4)	​	399	140,522	13.1 (11.8–14.6)
* Bad or very bad*	​	140	47,490	4.4 (3.6–5.3)	​	160	62,394	5.8 (4.9–7.0)
Importance of one’s health	48	​	​	​	32	​	​	​
* Lives without concern of health*	​	271	84,116	7.8 (6.8–8.9)	​	375	128,323	12.1 (10.8–13.5)
* Lifestyle to maintain health*	​	2,370	754,556	70.2 (68.3–72.0)	​	2,100	729,549	68.7 (66.7–70.6)
* Health consideration determines a lot of one’s life*	​	747	236,350	22.0 (20.4–23.7)	​	574	203,996	19.2 (17.6–20.9)
Health services utilisation								
Medical visit in the past year	0	​	​	​	2	​	​	​
* No*	​	455	140,564	12.9 (11.6–14.3)	​	704	241,346	22.5 (20.8–24.3)
* Yes*	​	2,981	951,249	87.1 (85.7–88.4)	​	2,375	830,857	77.5 (75.7–79.2)
Number of medical visits in the past year	26	​	​	​	8	​	​	​
* 0–1*	​	932	290,146	26.8 (25.1–28.6)	​	1,270	428,679	40.1 (38.0–42.1)
* 2–3*	​	995	306,715	28.3 (26.6–30.1)	​	803	283,337	26.5 (24.7–28.4)
* 4–5*	​	615	202,302	18.7 (17.2–20.3)	​	432	153,242	14.3 (12.9–15.9)
* *>5	​	868	283,564	26.2 (24.5–28.0)	​	568	204,741	19.1 (17.5–20.9)
Hospitalisation in the past year	13	​	​	​	16	​	​	​
* No*	​	3,033	954,614	87.9 (86.5–89.2)	​	2,711	939,640	88.0 (86.5–89.3)
* Yes*	​	390	131,376	12.1 (10.8–13.5)	​	354	128,388	12.0 (10.7–13.5)
*S*ecurity and social supports								
Social support (Oslo 3-item scale)^7^	147	​	​	​	124	​	​	​
* Low*	​	369	124,991	12.0 (10.7–13.4)	​	331	125,306	12.2 (10.8–13.7)
* Moderate*	​	1,451	465,479	44.7 (42.7–46.7)	​	1,353	469,239	45.7 (43.6–47.8)
* High*	​	1,469	451,208	43.3 (41.3–45.3)	​	1,273	432,511	42.1 (40.0–44.2)
Annual deductible	155	​	​	​	149	​	​	​
* CHF 300*	​	1,568	493,477	47.4 (45.3–49.4)	​	1,125	383,272	37.5 (35.5–39.6)
* CHF 500, 1,000 or 1,500*	​	1,088	350,390	33.6 (31.7–35.6)	​	977	337,215	33.0 (31.0–35.1)
* CHF 2,000 or 2,500*	​	625	197,881	19.0 (17.5–20.6)	​	830	300,677	29.4 (27.5–31.5)
Health insurance model^8^	63	​	​	​	63	​	​	​
* Ordinary or bonus*	​	1,597	517,446	48.2 (46.2–50.2)	​	1,530	541,946	51.6 (49.5–53.7)
* Managed care*	​	1,776	556,110	51.8 (49.8–53.8)	​	1,488	509,008	48.4 (46.3–50.5)
Last skin screening	8	​	​	​	4	​	​	​
* ≥ 5 years or never*	​	2,207	700,431	64.3 (62.4–66.2)	​	2,115	731,239	68.3 (66.3–70.2)
* < 5 years*	​	1,221	388,250	35.7 (33.8–37.6)	​	962	339,765	31.7 (29.8–33.7)
Last cervical screening	36	​	​	​	​	-	​	-
* ≥ 3 years or never*	​	1,018	324,786	30.1 (28.3–32.0)	​	-	-	​
* < 3 years*	​	2,382	753,296	69.9 (68.0–71.7)	​	-	-	-
Last breast screening	9	​	​	​	​	-	-	-
* ≥ 2 years or never*	​	1,696	554,692	51.0 (49.0–53.0)	​	-	-	-
* < 2 years*	​	1,731	532,945	49.0 (47.0–51.0)	​	-	-	-
Last colorectal screening	25	​	​	​	22	​	​	​
* Colonoscopy ≥ 10 years or FOBT ≥ 2 years or never*	​	1,899	599,268	55.4 (53.4–57.3)	​	1,683	596,538	56.1 (54.0–58.2)
* Colonoscopy < 10 years or FOBT < 2 years*	​	1,512	483,253	44.6 (42.7–46.6)	​	1,376	467,067	43.9 (41.8–46.0)
Last prostate screening	​	-	-	-	15	​	​	​
* ≥ 2 years or never*	​	-	-	-	​	1,832	647,406	60.6 (58.6–62.6)
* < 2 years*	​	-	-	-	​	1,234	420,543	39.4 (37.4–41.4)
Number of cancer screening within recommended intervals	25	​	​	​	17	​	​	​
* 0*	​	383	116,861	10.8 (9.6–12.1)	​	942	330,449	31.0 (29.1–33.0)
* 1*	​	746	245,691	22.7 (21.0–24.5)	​	1,000	355,957	33.4 (31.5–35.4)
* 2*	​	1,085	347,138	32.1 (30.2–34.0)	​	794	264,527	24.8 (23.1–26.7)
* 3*	​	858	272,319	25.2 (23.5–26.9)	​	328	114,121	10.7 (9.5–12.1)
* 4*	​	339	100,205	9.3 (8.2–10.4)	​	-	​	-
Concurrent utilisation	25	​	​	​	17	​	​	​
* No*	​	2,214	709,690	65.6 (63.7–67.4)	​	1,942	686,406	64.4 (62.4–66.4)
* Yes*	​	1,197	372,524	34.4 (32.6–36.3)	​	1,122	378,648	35.6 (33.6–37.6)

CI: confidence interval. FOBT: faecal occult blood test. CHF: Swiss Francs (1 CHF ≈1.07 Euro ≈1.25 US, Dollar as of January 2026).

1 Primary: compulsory school. Secondary: high school or apprenticeship. Tertiary education: university, university of applied sciences, or higher vocational education and training institutions.

2 Urban: urban municipality in a large, medium-sized or small conurbation, or outside a conurbation. Dense suburbs and rural centres: high-density or medium-density suburban municipality, or municipality in a rural centre. Rural: low-density suburban community, centrally located rural community, or outlying rural community.

3 Equivalised disposable income: net monthly income adjusted for the number of members in the household.

4 Inactive: <30 min of moderate physical activity or <1 time of intense activity per week. Partially active: 30–149 min of moderate physical activity or 1 time of intense activity per week. Sufficiently active: ≥150 min of moderate physical activity or ≥2 times intense physical activity per week.

5 Low risk: less than 40 g/day of pure alcohol for men and less than 20 g/day for women. Moderate risk: between 40 and 60 g/day of pure alcohol for men and between 20 and 40 g/day for women. High-risk: more than 60 g/day of pure alcohol for men and more than 40 g/day for women.

6 Underweight: body mass index less than 18.5 kg/m2. Normal weight: body mass index between 18.5 and 24.9 kg/m2. Overweight: body mass index between 25.0 and 29.9 kg/m2. Obese: body mass index equal or more than 30.0 kg/m2.

7 Low: score between 3 and 8. Moderate: score between 9 and 11. High: score between 12 and 14.

8 Bonus: Reduction in premium if no claims were made for 1 year. Managed care: health maintenance organisation centre, general practitioner, or medical advice hotline.

**FIGURE 1:**
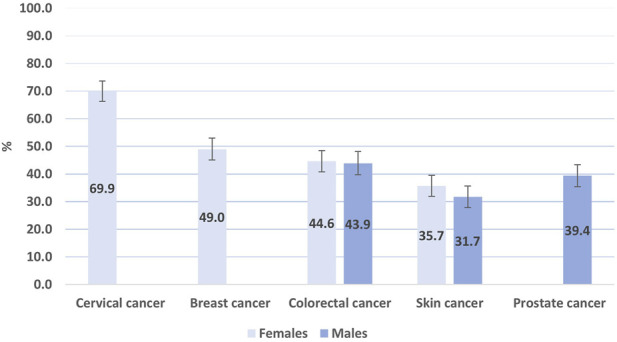
Proportion of self-reported cervical, breast, colorectal, skin and prostate cancer screening within recommended intervals, for female and male residents aged 50–69 (Swiss Health Survey, Switzerland, 2017).

Most females had undergone a screening test within recommended intervals for cervical cancer (69.9%, 95% CI: 68.0–71.7), whereas slightly fewer than half had undergone screening for breast (49.0%, 95% CI: 47.0–51.0) and colorectal (44.6%, 95% CI: 42.7–46.6) cancers (Figure 1). Less than half of males had undergone colorectal (43.9%, 95% CI: 41.8–46.0) and prostate (39.4%, 95% CI: 37.4–41.4) cancer screening within the recommended intervals. Approximately one in three females (35.7%, 95% CI: 33.8–37.6) and males (31.7%, 95% CI: 29.8–33.7) had a skin examination in the last 5 years.

**FIGURE 2:**
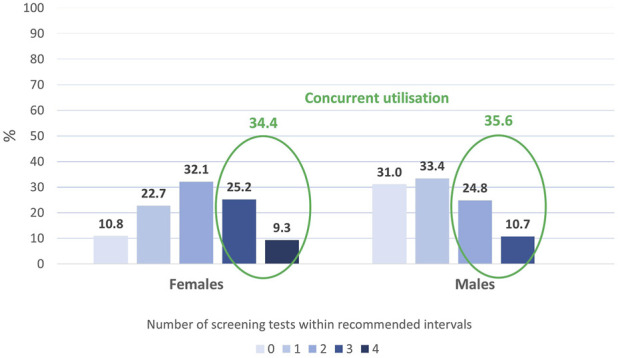
Proportion of self-reported cancer screening tests by number of tests performed within recommended intervals, for female and male residents aged 50–69 (Swiss Health Survey, Switzerland, 2017).

The proportion of concurrent screening was similar between females (34.4%, 95% CI: 32.6–36.3) and males (35.6%, 95% CI: 33.6–37.6) (Figure 2).

Table 2 shows results from the multivariable models. Concurrent utilisation of cancer screenings among females increased with age (OR per year = 1.03). Females living in French- or Italian-speaking Switzerland were approximately 2.3 times more likely to concurrently use cancer screenings than those living in the German-speaking region. Females with tertiary education and those with several medical visits in the past year were also more likely to report concurrent utilisation. The odds of concurrent utilisation increased 2.7 times for females with 2-3 medical visits, and approximately 4.7 times for those with 4 or more visits.

**TABLE 2:** Adjusted associations between concurrent utilisation of cancer screening tests and each covariate, for female and male residents aged 50–69 (Swiss Health Survey, Switzerland, 2017).

​	Females (weighted count = 1,081,933)^*^		Males (weighted count = 1,064,901)^**^	
​	aOR (95% CI)	p-value Wald test	aOR (95% CI)	p-value Wald test
Socio-demographics				
Age (increase per age-year)	1.03 (1.01–1.05)	<0.001	1.07 (1.05–1.08)	<0.001
Education^1^	​	0.01	​	0.23
* Primary*	0.87 (0.66–1.14)	​	​	​
* Secondary*	1.00 (reference)	​		​
* Tertiary*	1.36 (1.09–1.69)	​		​
Marital status	​	<0.01	​	0.76
* Single*	0.76 (0.56–1.05)	​	​	​
* Married*	1.00 (reference)	​		​
* Divorced or separated*	0.72 (0.56–0.92)	​		​
* Widowed*	0.56 (0.38–0.81)	​		​
Occupation	​	0.83	​	0.85
* Economically inactive*	​	​	​	​
* Unemployed or homemaker*		​		​
* Employed*		​		​
Nationality	​	0.04	​	0.21
* Swiss*	1.00 (reference)	​	​	​
* Northern and western European*	0.68 (0.41–1.13)	​		​
* South-western European*	0.73 (0.47–1.12)	​		​
* Eastern and south-eastern European*	0.68 (0.36–1.29)	​		​
* Non-European*	0.06 (0.00–0.65)	​		​
Linguistic region	​	<0.001	​	0.16
* German-speaking*	1.00 (reference)	​	​	​
* French-speaking*	2.25 (1.85–2.75)	​		​
* Italian-speaking*	2.28 (1.64–3.17)	​		​
Region of residence^2^	​	0.17	​	0.32
* Urban*	​	​	​	​
* Dense suburbs and rural centres*		​		​
* Rural*		​		​
Household type	​	0.34	​	0.01
* Single individual*	​	​	0.79 (0.60–1.04)	​
* Couple without children*		​	1.00 (reference)	​
* Couple with children*		​	1.22 (0.97–1.55)	​
* Single parent with children*		​	0.65 (0.35–1.17)	​
* Multi-family household or non-family household with several individuals*		​	0.53 (0.28–1.01)	​
Equivalised disposable income^3^	​	0.05	​	<0.001
* < CHF 3,000*	0.80 (0.61–1.05)	​	1.17 (0.86–1.60)	​
* CHF 3,000–4,499*	1.00 (reference)	​	1.00 (reference)	​
* CHF 4,500–5,999*	0.96 (0.74–1.26)	​	1.48 (1.12–1.94)	​
* ≥ CHF 6,000*	1.21 (0.93–1.57)	​	2.25 (1.74–2.92)	​
Lifestyle	​	​	​	​
Physical activity^4^	​	0.87	​	0.08
* Inactive*	​	​	0.66 (0.45–0.97)	​
* Partially active*		​	0.89 (0.68–1.15)	​
* Sufficiently active*		​	1.00 (reference)	​
Fruits and/or vegetables consumption	​	0.03	​	0.68
* < 5 days per week*	0.78 (0.49–1.26)	​	​	​
* 0–2 portions per day ≥ 5 days per week*	0.79 (0.63–0.99)	​		​
* 3–4 portions per day ≥ 5 days per week*	1.00 (reference)	​		​
* ≥ 5 portions per day ≥ 5 days per week*	1.12 (0.90–1.39)	​		​
Alcohol consumption^5^	​	0.54	​	0.14
* Abstainer*	​	​	0.92 (0.66–1.29)	​
* Low risk*		​	1.00 (reference)	​
* Moderate or high risk*		​	0.65 (0.43–1.00)	​
Smoking status	​	0.03	​	0.24
* Never*	1.00 (reference)	​	​	​
* Ex-smoker*	1.11 (0.90–1.38)	​		​
* Smoker*	0.79 (0.63–1.00)	​		​
Health	​	​	​	​
Body mass index^6^	​	0.41	​	0.28
* Underweight*	​	​	​	​
* Normal weight*		​		​
* Overweight*		​		​
* Obese*		​		​
Chronic disease or long-term health problem	​	0.36	​	0.07
* No*	​	​	1.00 (reference)	​
* Yes*		​	0.82 (0.67–1.01)	​
Cancer in the past year	​	0.15	​	0.39
* No*	​	​	​	​
* Yes*		​		​
Self-reported health	​	1.00	​	0.44
* Very good or good*	​	​	​	​
* Medium*		​		​
* Bad or very bad*	​	​		​
Importance of one’s health	​	0.13	​	0.01
* Lives without concern of health*	​	​	0.62 (0.45, 0.85)	​
* Lifestyle to maintain health*		​	1.00 (reference)	​
* Health consideration determines a lot of one’s life*		​	0.80 (0.63, 1.02)	​
Health services utilisation	​	​	​	​
Number of medical visits in the past year	​	<0.001	​	<0.001
* 0–1*	1.00 (reference)	​	1.00 (reference)	​
* 2–3*	2.69 (2.08, 3.49)	​	2.89 (2.27, 3.68)	​
* 4–5*	4.86 (3.65, 6.46)	​	2.68 (1.99, 3.61)	​
* > 5*	4.65 (3.54, 6.11)	​	3.25 (2.44, 4.32)	​
Hospitalisation in the past year	​	0.06	​	0.89
* No*	1.00 (reference)	​	​	​
* Yes*	0.76 (0.56, 1.02)	​		​
*S*ecurity and social supports	​	​	​	​
Social support (Oslo 3-item scale)^7^	​	0.01	​	0.91
* Low*	0.74 (0.54, 1.03)	​	​	​
* Moderate*	1.00 (reference)	​		​
* High*	1.19 (0.98, 1.44)	​		​
Annual deductible	​	0.59	​	<0.01
* CHF 300*	​	​	1.00 (reference)	​
* CHF 500, 1,000 or 1,500*		​	0.67 (0.53, 0.85)	​
* CHF 2,000 or 2,500*		​	0.43 (0.26, 0.72)	​
Health insurance model [8]	​	0.82	​	0.83
* Ordinary or bonus*	​	​	​	​
* ssManaged care*		​		​

aOR: adjusted odds ratio. CI: confidence interval. CHF: Swiss Francs (1 CHF ≈1.07 Euro ≈1.25 US, Dollar as of January 2026).

^*^ Of 3,436 females, 3,410 (99.2%) were included in the final model. Twenty-six (0.8%) were excluded (25 had missing data for the outcome; 1 had missing data for smoking and including it as a separate category in multivariable analysis led to data sparsity).

^**^ Of 3,081 males, 3,063 (99.4%) were included in the final model. Eighteen (0.6%) were excluded (17 had missing data for the outcome; 1 had missing value for household type and including it as a separate category in multivariable analysis led to data sparsity).

1 Primary: compulsory school. Secondary: high school or apprenticeship. Tertiary education: university, university of applied sciences, or higher vocational education and training institutions.

2 Urban: urban municipality in a large, medium-sized or small conurbation, or outside a conurbation. Dense suburbs and rural centres: high-density or medium-density suburban municipality, or municipality in a rural centre. Rural: low-density suburban community, centrally located rural community, or outlying rural community.

3 Equivalised disposable income: net monthly income adjusted for the number of members in the household.

4 Inactive: <30 min of moderate physical activity or <1 time of intense activity per week. Partially active: 30–149 min of moderate physical activity or 1 time of intense activity per week. Sufficiently active: ≥150 min of moderate physical activity or ≥2 times intense physical activity per week.

5 Low risk: less than 40 g/day of pure alcohol for men and less than 20 g/day for women. Moderate risk: between 40 and 60 g/day of pure alcohol for men and between 20 and 40 g/day for women. High-risk: more than 60 g/day of pure alcohol for men and more than 40 g/day for women.

6 Underweight: body mass index less than 18.5 kg/m2. Normal weight: body mass index between 18.5 and 24.9 kg/m2. Overweight: body mass index between 25.0 and 29.9 kg/m2. Obese: body mass index equal or more than 30.0 kg/m2.

7 Low: score between 3 and 8. Moderate: score between 9 and 11. High: score between 12 and 14.

8 Bonus: Reduction in premium if no claims were made for 1 year. Managed care: health maintenance organisation centre, general practitioner, or medical advice hotline.

Divorced or separated females (aOR=0.72, 95% CI: 0.56–0.92) and widows (aOR=0.56, 95% CI: 0.38–0.81) were less likely to report concurrent utilisation than married females. Non-European females were also less likely to report concurrent utilisation (aOR=0.06, 95% CI: 0.00–0.65) compared with Swiss females, albeit this finding was based on a small number of non-European participants (Table 1). Concurrent utilisation was approximately 20% lower among females consuming 0–2 portions of fruits and/or vegetables per day compared with those consuming 3-4 portions (aOR=0.79, 95% CI: 0.63–0.99), and among smokers compared with non-smokers (aOR=0.79, 95% CI: 0.63–1.00). Females with an EDI below CHF 3,000 were less likely to be concurrent screening users than those with an EDI above CHF 6,000. Concurrent utilisation also tended to be lower among those who had been hospitalised in the past year and among those with lower levels of social support.

Among males, concurrent utilisation of cancer screenings increased markedly with age (OR per year = 1.07). Household type was associated with concurrent utilisation with suggestive evidence that males living alone, as a single parent, or in a multi-family household were less likely concurrent users, whereas those living as a couple with children were more likely to be concurrent users. Males with an EDI of CHF 4,500 or higher were more likely to use cancer screening concurrently than those with an EDI of CHF 3,000–4,499. The odds of concurrent utilisation increased approximately two-to-three-fold among those with several medical visits in the past year.

Males who reported no concern for their health had lower odds of concurrent utilisation (aOR=0.62, 95% CI: 0.45–0.85). Regarding lifestyle factors, concurrent utilisation was 34% lower among physically inactive males compared with those who were sufficiently active (aOR=0.66, 95% CI: 0.45–0.97), and among those with moderate or high-risk alcohol consumption compared with those at low risk (aOR=0.65, 95% CI: 0.43–1.00). There was also suggestive evidence that males with a chronic disease were less likely to use cancer screening concurrently (aOR=0.82, 95% CI: 0.67–1.01). Compared with those with the lowest annual deductible of CHF 300, males with a higher deductible were less likely to be concurrent users.

## Discussion

We estimated that one in three Swiss individuals reported concurrent utilisation of recommended cancer screenings. Previous nationwide studies investigating concurrent utilisation of cancer screenings have reported a range from 27% to 55% of individuals being up-to-date with all recommended screenings [41, 45, 46, 48, 49], likely reflecting differences in study design, screening culture, and screening delivery. Overall, studies based on data from organised programmes reported higher proportions of concurrent screenings, up to 55% in Denmark [41, 49]. Most previous studies focused on females, and the full utilisation of breast, cervical, and colorectal cancer screenings. Although our outcome reflected near-complete concurrent utilisation, the results are largely comparable, as skin cancer screening, which is rarely included in international studies, has no formal recommendation in Switzerland. The proportion of concurrent utilisation in Switzerland was comparatively lower than in countries with more extensive organised cancer screening programmes, but aligns with recent national surveys investigating opportunistic and organised cancer screening, reporting 27% in Japan [45] and 42% among females and 29% among males in the United States [49].

One of the main findings was that factors associated with concurrent utilisation differed between sexes. The few common factors were increasing age, higher income and several medical visits in the past year. A substantially greater increase per year of age was observed in males than in females aged between 50–69. Younger individuals, particularly males, may perceive themselves as being at lower risk of cancer due to their age, or they may be harder to sensitise because of less frequent contact with healthcare providers. Income was significantly associated with concurrent utilisation, especially among males, suggesting that preventive health services may be a lower priority for males and are more likely to be used only by those with higher salaries. However, these results should be interpreted with caution because of the large number of missing income values. Of other sociodemographic factors, higher education, marital status, nationality, and linguistic region were all positively associated with concurrent utilisation, but only among females. In the Netherlands, higher levels of participation were observed among females with higher median household income [46]. Previous studies have also shown that full utilisation of cancer screenings was associated with higher educational attainment [45, 49]. Concurrent utilisation of screening tests was also associated with household type among males. This suggests that males may be more inclined to undergo screening when living with someone who does, especially a female, who is more likely to engage in cancer screening [59–61]. Additionally, a recent U.S. study reported a positive association between living with someone and being up-to-date with screening recommendations [62]. Regarding regional variations, higher utilisation of cancer screening in French- and Italian-compared with German-speaking Switzerland was observed separately for colorectal, breast and prostate cancer screening [50, 51, 63]. Greater use of health services and the broader availability of organised screening programmes (breast, colorectal) in Latin-speaking Switzerland likely contributed to these regional differences.

Among lifestyle factors, higher consumption of fruits and/or vegetables and current non-smoking status were suggestively associated with concurrent utilisation among females, whereas higher levels of physical activity and low-risk alcohol consumption were associated with concurrent use of cancer screening among males. In Japan, female smokers were also less likely to participate in cancer screening [45]. In a regional study in France, lower concurrent utilisation of organised breast and colorectal cancer screenings, as well as opportunistic cervical cancer screening, were associated with low levels of physical activity and smoking [43]. The association between unhealthy lifestyle factors and lower concurrent utilisation may reflect a low degree of health concern. Indeed, males without concern for their health were less likely to use cancer screening concurrently in Switzerland. Furthermore, having a chronic disease or a long-term health problem was associated with an 18% reduction (95%CI: −33%, +1%) in the odds of concurrent utilisation among males. In England, a nationwide case-control study among females reported that concurrent participation in all three recommended cancer screening programmes was higher in areas where general practices had more patients with a chronic disease [48]. In contrast, a regional study found that participation, although not concurrent, in all three programmes was more likely to be higher in females with few comorbidities or a history of benign neoplasms [47]. Overall, these results highlight the potentially ambivalent effect of chronic conditions on preventive care. Although individuals with chronic diseases have more frequent interactions with the healthcare system and may thus be more exposed to cancer screening recommendations, the demands of managing their illness can create competing priorities that reduce their likelihood of participating in screening. Accounting for the severity and type of comorbidity in future studies may help clarify the association between comorbidities and concurrent cancer screening use.

Several medical visits in the past year were strongly associated with concurrent utilisation in both sexes. Our findings corroborate those from a French survey, which reported that females who visited a gynaecologist or general practitioner in the past year were more likely to participate in at least two of three screenings for breast, colorectal and cervical cancer [43]. This underscores the key role of healthcare providers in promoting cancer screening and aligns with our observation of higher concurrent utilisation in the Latin Swiss regions, where general practitioners and gynaecologists facilitate access to organised screening programmes. As most cancer screenings involve medical visit(s), the observed association between visit frequency and concurrent screening utilisation may be artificially amplified, despite considering only visits in the past year.

Our results indicated that low social support may reduce the likelihood of concurrent screening among females, echoing recent findings of a positive association between social support and cancer screening participation in the U.S. [62]. An annual deductible above the legal minimum of CHF 300 was associated with decreased odds of concurrent utilisation among males. In contrast, an earlier Swiss study using 2012 SHS data reported no differences in patterns of screening utilisation for prostate, colorectal, or skin cancer according to annual deductible [32]. However, among females, higher deductibles were associated with lower odds of undergoing breast and cervical cancer screening, but not colorectal or skin cancer screening [32]. Higher annual deductibles are likely correlated with higher income and socioeconomic status.

In Switzerland, concurrent utilisation of cancer screening appears to be primarily influenced by sociodemographic factors among females and lifestyle and health factors among males. These findings have potentially important implications for cancer prevention. Interventions targeting females with persistently low screening rates due to sociodemographic characteristics and males with unhealthy lifestyle, might enhance screening uptake. Furthermore, sensitizing younger age groups (i.e., around 50 years) about benefits of regular cancer screening is important, as cancer risk strongly increases with age.

### Strengths

Our study provides the first insights into patterns of concurrent cancer screening utilisation in the Swiss population aged 50–69 and explores multiple multidimensional factors likely to be associated with screening practices, including rather rare variables that approximate individuals’ health preferences and attitudes toward their health. Results from this randomly selected, nationwide, stratified sample are representative of the Swiss population, and weighting reduces potential selection bias due to non-response. Finally, structured interviews conducted by trained professionals ensure the quality and consistency of SHS data.

### Limitations

The SHS was not specifically designed for the purposes of our research (e.g., questions on prior cancer diagnosis were limited to the past year, with no information on cancer type, preventing any assessment of associations with related cancer screenings). The cross-sectional design poses measurement limitations. Substantial heterogeneity in recommended screening intervals across cancer types - from biennial to decennial - may introduce differential recall bias, such that observed associations may not reflect long-term adherence. Because screening intervals are designed to align with the natural history and biological progression of each cancer, a cross-sectional snapshot may not accurately capture the sustained utilisation required for clinical efficacy. Potential response bias may arise from self-reported data. Social desirability bias could have led to overestimation of healthy behaviours and underestimation of less healthy behaviours. However, the estimated proportion of cancer screenings utilisation closely matched data from organised programmes [64], and numerous indicators of health behaviour corresponded to estimates from other sources [65] (p.30,p.49), [66] (p.35). While male and female results are discussed in relation to one other, direct comparison is limited by differences in recommended screenings, accessibility, statistical models, and definition of concurrency. Under our definition, men can achieve concurrency through two easily accessible screenings conducted during routine medical visits (e.g., prostate and skin), whereas women must complete three screenings, including at least one that requires a scheduled procedure (e.g., breast or colorectal) to meet the criteria for concurrent utilisation. Finally, the results may not be fully representative of the Swiss population for factors unadjusted by survey weights (e.g., income) due to missing data. Multiple imputation was not applied because missingness for key socioeconomic variables was likely not missing at random, suitable auxiliary variables to support robust imputation were limited, and the complex survey design and weighting structure would have required strong reliance on untestable imputation assumptions. Given the absence of a significant association between missing categories and the outcome, except for annual deductible among men, which suggests minimal bias attributable to missing data, missing values were handled as a separate category to ensure transparency and preserve sample size.

Further studies are warranted to better understand the mechanisms underlying different user profile. In particular, reasons for adherence with one screening recommendation but not others, and the mediating pathways linking unhealthy behaviours, chronic disease, and low health concern need to be explored. Given the temporal instability of screening behaviours, findings from a single survey may not fully capture the sustained adherence required for clinical efficacy and should be interpreted with caution when informing policy changes in cancer screenings.

## Data Availability

The data that support the findings of this study are available from the FSO, but restrictions apply to the availability of these data, which were used under license for the current study, and thus are not publicly available. The anonymised data can be accessed upon a formal request to the FSO (https://www.bfs.admin.ch/bfs/fr/home/statistiques/sante/enquetes/sgb.html#accs-aux-donnes-anonymises).
